# Highly sex specific gene expression in Jojoba

**DOI:** 10.1186/s12870-023-04444-z

**Published:** 2023-09-19

**Authors:** Bader Alsubaie, Ardashir Kharabian-Masouleh, Agnelo Furtado, Othman Al-Dossary, Ibrahim Al-Mssallem, Robert J. Henry

**Affiliations:** 1 Queensland Alliance for Agriculture and Food Innovation, University of Queensland, Brisbane, 4072 Australia; 2 College of Agriculture and Food Sciences, King Faisal University, 36362 Al Hofuf, Saudi Arabia; 3 ARC Centre of Excellence for Plant Success in Nature and Agriculture, University of Queensland, Brisbane, 4072 Australia

**Keywords:** Jojoba, Chromosomes, Differentially expressed genes, RNA-Seq, Flower, Novel genes, Dioecious plants, *Simmondsia chinensis*

## Abstract

**Background:**

Dioecious plants have male and female flowers on separate plants. Jojoba is a dioecious plant that is drought-tolerant and native to arid areas. The genome sequence of male and female plants was recently reported and revealed an X and Y chromosome system, with two large male-specific insertions in the Y chromosome.

**Results:**

A total of 16,923 differentially expressed genes (DEG) were identified between the flowers of the male and female jojoba plants. This represented 40% of the annotated genes in the genome. Many genes, including those responsible for plant environmental responses and those encoding transcription factors (TFs), were specific to male or female reproductive organs. Genes involved in plant hormone metabolism were also found to be associated with flower and pollen development. A total of 8938 up-regulated and 7985 down-regulated genes were identified in comparison between male and female flowers, including many novel genes specific to the jojoba plant. The most differentially expressed genes were associated with reproductive organ development. The highest number of DEG were linked with the Y chromosome in male plants. The male specific parts of the Y chromosome encoded 12 very highly expressed genes including 9 novel genes and 3 known genes associated with TFs and a plant hormone which may play an important role in flower development.

**Conclusion:**

Many genes, largely with unknown functions, may explain the sexual dimorphisms in jojoba plants and the differentiation of male and female flowers.

**Supplementary Information:**

The online version contains supplementary material available at 10.1186/s12870-023-04444-z.

## Background

Dioecious plant species have male and female flowers on separate individuals and represent only 6% of flowering plant species [1]. The evolutionary mechanisms that may explain the development of dioecious species have been described in detail [2, 3]. The dioecious species either evolved from hermaphrodites by termination of the androecium or gynoecium (e.g., *Silene latifolia*) or the differentiation of the sexes in the plants occurs before the initiation of the reproductive part of the flowers (e.g., in *Populus*). Sex determination is affected by environmental factors [4], and the genetic systems [5] in some dioecious plants. Understanding the differences between male and female plants in dioecious species will assist the development of sex markers and provide a strong genomic foundation to understanding sex differentiation through reproductive flower organ development. RNA sequencing (RNA-Seq) is a powerful technology used in transcriptome analysis to obtain a global understanding of biological pathways because it is an efficient approach for gene expression analysis at the transcriptome level [6]. The RNA-seq data could be used in differentially expressed genes (DEGs) analysis to provide a molecular basis for revealing differences at the transcriptional levels between male and female flowers in some dioecious plants. In *Cannabis sativa*, RNA-Seq was applied to determine differentially expressed genes that were associated with the development of male or female plants. The results showed over 10,500 DEGs, of which around 200 were linked with male or female flower development [7]. In asparagus, around 4876 DEGs were determined as the likely sex-determining stage of female and male flower buds [8]. In dioecious spinach (*Spinacia oleracea*L.) RNA-Seq analysis was used on male and female spinach flowers to understand the sex differentiation mechanism, revealing a total of 2965 DEGs between male and female flowers [2].

Jojoba (*Simmondsia chinensis*) is dioecious and drought resistant plant native to North America [9]. The jojoba male has a united inflorescence cluster composed of 7 to 36 individual flowers. The female flower is (8–14 mm long) and is larger than the individual male flowers (only 3–5 mm) and is produced individually on the plant [10]. Many efforts have been made to determine the genetic basis of sexual dimorphism in jojoba plants using cytological methods and identified sex-related genetic markers from other plants [11, 12]. However, the sequencing of the male and female jojoba genomes revealed a XY chromosome system, in which chromosome 9 was the sex chromosome [13]. The Y chromosome was found to have two large male-specific insertions (Y1 and Y2 with a total length of 10 Mb) [13]. We now report on RNA Seq analysis of the expression of genes from male and female jojoba flowers including the genes in the sex specific chromosome regions. RNA-Seq analysis [14, 15] was recently used in jojoba plants to determine the expression of salt-related genes [16] and genes involved in storage lipid synthesis in the seeds [17]. Annotation will be aided by transcriptome sequencing utilizing both short and long read technologies. Application of RNA-Seq technology to measuring the expression of jojoba genes from male and female flowers will give information on how genes are regulated and reveal new genes that are involved in flower development, growth, and reproduction. The goal of this study was to perform RNA-Seq and differential gene expression analysis to investigate gene expression during flower development and explore the difference between male and female plants. Understanding gene expression in a plant for which the genome has been fully sequenced may improve our understanding of the specific development of male and female flowers and the evolution of dioecious plants.

## Results

### Functional annotation for the reference of RNA-Seq analysis

The male genome sequence that has been previously reported [13] was annotated specifically for this study using RNA Seq data from leaves and flowers to support the annotation. The coding sequences (CDS) of the male genome was chosen to be the reference for the RNA-Seq analysis as the male also has a female (X) chromosome, and as a result all male and female genes are present in the male reference. A total of 43,807 CDS were detected using the GenSAS annotation pipeline [18] and used as a reference for RNA-Seq analysis. The completeness of the annotation was confirmed using Benchmarking Universal Single-Copy Orthologs (BUSCO) analysis. The complete BUSCOs count was 249 (97.6%) using the eukaryota_odb10 lineage dataset with 217 (85.1%) and 32 (12.5%) single and duplicated copies, respectively (Figure S1A). The reference of RNA-Seq was functionally annotated using different databases from the OmicsBox pipeline to investigate the function of the jojoba genes.

Of the 43,804 CDS, 25,435 transcripts had matches with the BLAST using the non-redundant protein sequences, of the Viridiplantae database while 18,117 transcripts had no matches (no hit) in the BLAST database. In the InterProScan (IPS) database, 33,150 transcripts were detected with matches, while only 10,657 did not result in a match (No IPS) and 15,671 transcripts had GOs. Of the 15,671 transcripts with GOs, protein kinase domains (IPR000719, 875 matches) received most of the hits, followed by zinc finger, RING type (IPR001841, 408 matches), RNA recognition motif domain (IPR000504, 472 matches), serine-threonineltyrosine-protein kinase (IPR001245, 334 matches), domain of unknown function DUF4283 (IPR025558, 221 matches) (Figure S2).

GO terms are generally divided into three main categories: biological process, molecular function, and cellular component. The molecular function (MF, 169,74 transcripts) had the highest number of transcripts compared to the biological process (BP, 136,82 transcripts) and the cellular component (CC, 12,308 transcripts). Under BP, the highest three transcripts were involved in the following processes: cellular process (11,631 transcripts), metabolic process (9979 transcripts), organic substance metabolic process (9104 transcripts). The top three transcripts in MF were binding (11,540 transcripts), followed by catalytic (9328 transcripts) and organic cyclic compound binding (7098 transcripts). Finally, the top three families in CC were cellular anatomical entity (12,162 transcripts) followed by intracellular anatomical structure (8118 transcripts) and organelle (6787 transcripts) (Figure S1B). The distribution of enzyme codes was further investigated, indicating that the enzyme code class of transferases had the largest number with 3741 transcripts followed by hydrolases with 2909 transcripts and oxidoreductases 1475 transcripts (Figure S1C). The functional annotation of the jojoba CDS reference data revealed a close relationship between jojoba and different plant species based on the origin of the BLAST hit sequences. The highest number of BLAST hits was associated with beetroot (*Beta vulgaris subsp. vulgaris*) with 4430 hits followed by quinoa (*Chenopodium quinoa*) with 3987 matches, spinach (*Spinacia oleracea*) 2464 matches. These three plant species share the same order, *Caryophyllales,* with jojoba (*Simmondsia chinesis*) (Figure S1D).

### RNA-Seq analysis

The RNA-Seq of different flower stages of both male and female jojoba plants were used to compare the gene expression between male and female jojoba plants to understand the plant's response during flowering. The raw RNA reads from male and female samples were quality trimmed at 0.01 score. The number of raw reads for the 15 male samples before trimming ranged between 84,120,716 to 105,964,768 reads whereas the number of the reads after trimming at 0.01 became 82,252,616 to 103,688,687 reads. The number of raw reads for the 15 female RNA samples ranged between 86,429,200 to 106,110,096 reads and it became 84,344,187 to 103,893,501 after trimming at 0.01 score. The trimmed reads of male and female samples were selected for RNA-Seq analysis (Table S1). The combined RNA-Seq reads of the male and female samples were mapped against the reference CDS male genome. The results showed the percentage of reads mapping ranging between 52 to 55% against the reference for the five male groups respectively. The mapping percentage ranged between 51 to 55% for the five female groups (Figure S3). The mapping reads of the male and female groups were collected and used for DE analysis. A principal component analysis (PCA) was conducted for the five male and female groups. The principal component 1 explained 51.9% whereas second principal component explained 12.8%. The five female groups showed a tight cluster with each other whereas the five male groups showed a cluster in R1MS2, S3, S4, R2MS2, S3, S4, and R3MS2, S3, S4 and with a small separation from R4M S2, S3, S4 and one outlier group R5M S2, S3, S4 (Figure S4).

### Differentially expressed transcripts in male relative to female flowers in Jojoba

The comparison of the male combined group (treatment group) against the female combined group (control group) shed more light on the genes that were differentially expressed in the male and female groups. A total of 16,923 significantly differentially expressed genes (DEG) (FDR *p* < 0.01) were identified using DE analysis between the flowers of male and female jojoba plants which represented 40% of the annotated genes in the genome using CLC-GWS (Table 1).

**Table 1:** Differential expressed genes (DEGs) in the male relative to female flowers in jojoba plant

Component	Total number of annotated genes	Percentage of genes %	Number of DEGs		Total number of DEGs	Percentage of Genes differentially expressed
			**Up**	**Down**		
**Whole genome**	41,645	100	8,938	7,985	16,923*	40.6%
**Chromosome Y**	1,616	3.9%	510	460	970	60.0%

16,923* significantly differentially expressed genes (DEG) (FDR *p* < 0.01)

### Functional classification of DEGs

The up-regulated DEGs for the three GO categories BP, CC and MF were obtained (Figure S5). The three GO terms for down regulated DEGs were captured (Figure S6). The most highly differentially expressed transcripts associated with the MF were binding followed by catalytic activity and organic cyclic compound binding (Figure S6).

### KEGG pathways analysis

KEGG pathways analysis was carried out to categorise and annotate DEGs up and down regulated in the flowers of the jojoba plants using an OmicsBox pipeline. A total of 2515 pathways from the Reactome database and 296 pathways from the KEGG database were identified from 8921 up regulated DEGs (Table S2). The pathways with the largest number of DEGs were plant hormone signal transduction (132 seqs), plant-pathogen interaction (101 seqs), NOD-like receptor signaling pathways (100 seqs), MAPK signalling pathways (94 seqs). A total of 3193 pathways from Reactome database and 307 pathways KEGG database were captured from 7985 down-regulated DEGs (Table S2). The pathways associated with largest number of DEGs were Ribosome (255 seqs), Spliceosome (87 seqs), Biosynthesis of cofactors (83 seqs), and RNA transport (74 seqs) Table S2).

### DEGs associated with flower in Jojoba

Transcription factors (TFs) are proteins which play an important role in regulating gene expression and can act by enhancing or repressing the rate of transcription. They can control cellular pathways in response to abiotic and biotic stimuli, and affect floral organ and pollen development [19, 20]. Among the DEGs, 280 TFs were differentially expressed in male relative to female flowers. These genes are from six TF families, of which MYB (including 69 DEGs), ERF (including 50 DEGs), bHLH (including 47 DEGs), NAC (including 41 DEGs), WRKY (including 38 DEGs), and MADS-box (including 35 DEGs) (Table S3). Plant hormones play an important regulatory role in both plant development and responses. They can affect seed germination, flowering time, the sex of the flowers, and response to abiotic stress which can enhance drought and salt tolerance in plants [21]. There were 135 DEGs involved in hormone pathways; of these DEGs, 73 were involved in auxin pathway, 19 were involved in gibberellin pathway, 17 were involved in abscisic acid pathway, 12 were involved in cytokinin pathway, 12 were involved in salicylic acid pathway and only 2 were involved in jasmonic acid pathway (Table S4).

### Top ten up-down regulated (log fold change) associated with sex differentiation in Jojoba

The significance differentially expressed transcripts/genes at FDR corrected *p* value < 0.01; from the comparison of the male and female groups revealed 16,923 significantly DEGs. The top ten up and down regulated DEGs were selected and investigated for any genes that might be associated with flower. The highest fold change of the up regulated DEGs was 530,000 and the two most differentially expressed genes had no BLAST results and the other eight DEGs matched with known genes. Among the top 10 DEGs (up regulated), three were associated with pollen (cytochrome P450 704B1) [22], Defensin-like protein (DEFL) [23], and bidirectional sugar transporter SWEET5 [24], and three DEGs were linked with lipid ligand binding (LTP); nonspecific lipid transfer proteins from pollen (Plant lipid transfer protein/Par allergen) [25], non-specific lipid-transfer protein 1-like [26] and putative plant lipid transfer protein/Par allergen [27] (Table 2).

**Table 2:**
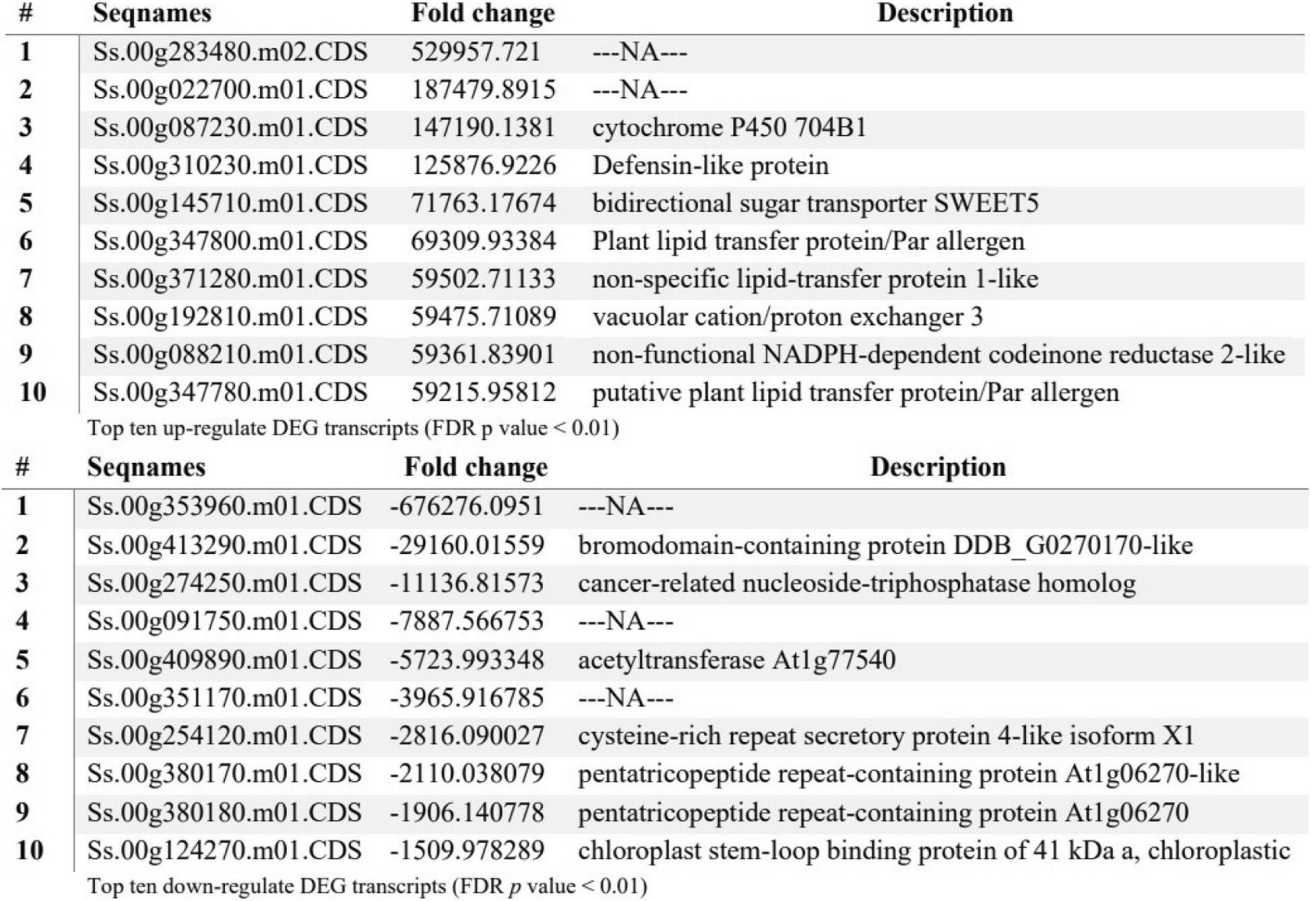
Top ten up and down regulated differentially expressed genes associated with flowers based on fold change of Jojoba plants

The most down-regulated gene had a fold change number of -676,276. Three of the DEGs had no blast results and the other seven DEGs matched with known genes. In *Arabidopsis thaliana*, the CYP704B1 gene is involved in pollen wall development [22] and the CYP704B1 gene was the third most down-regulated gene (Table 2).

### Expression of chromosome 9 including male-specific genes in Y1 and Y2 regions

**Fig. 1:**
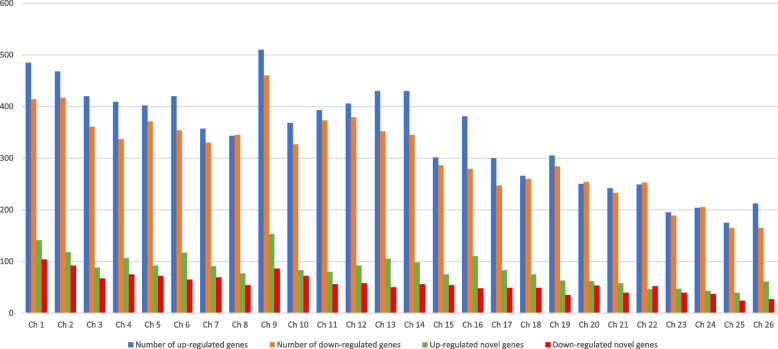
Distribution of the up and down including novel regulated genes on the male chromosome level assembly. The table shows the number of up-regulated genes (blue column), number of down-regulated genes (orange column), number of up-regulated novel genes (green column), and number of down-regulated novel genes (red column) across the chromosome level assembly. Chromosome 9 has the highest number of up (510), down (460) regulated genes, and up-regulated novel genes (153) among all chromosomes. The highest down-regulated genes linked with chromosome 1 (104)

The up and down DEGs were distributed on the male chromosome level assembly. The result revealed that the chromosome with the highest number of up-regulated DE was chromosome 9 which is considered to be the Y sex chromosome in jojoba [13] (Fig. 1). Out of the 8938 up-regulated genes, (24%) 2189 genes were novel, and their sequences length ranged from 185 to 663 bp. The largest number of novel transcripts were also linked to chromosome 9 (Fig. 1). The 7985 down-regulated genes were also aligned to the chromosome level assembly and showed the highest number of DE linked to chromosome number 9 (Fig. 1). Out of the 7985 genes, (18%) 1483 genes were novel genes with sizes ranging from 149–2412 bp. The highest number of novel genes was linked to chromosome 1 (Fig. 1).

**Fig. 2:**
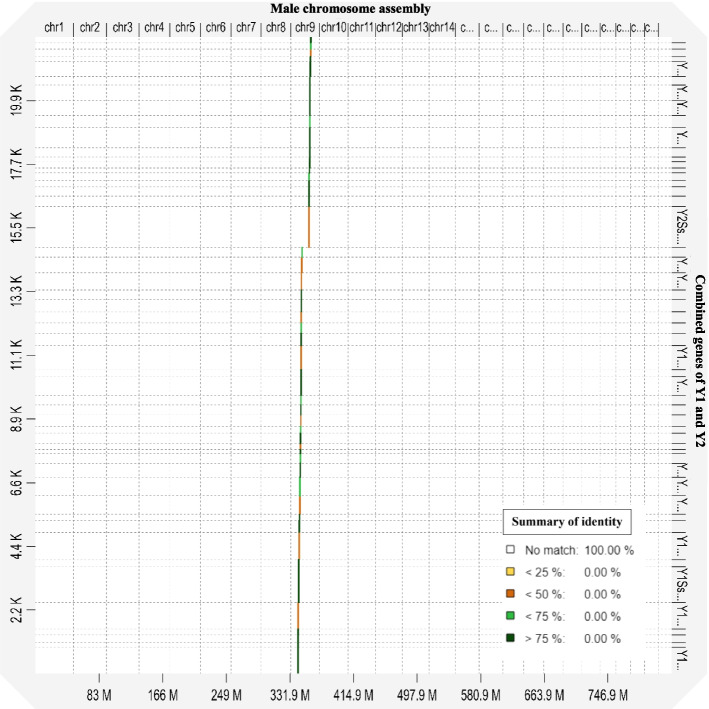
The X axis showed the male chromosome assembly and the Y axis displayed combined CDS of Y1 and Y2. A total of 51 male-specific genes (CDS) mapped only to chromosome 9 of the male genome, illustrating unique male-specific genes that are not found in the female genome. The colour of sequences displayed the percentage of match to the reference

**Fig. 3:**
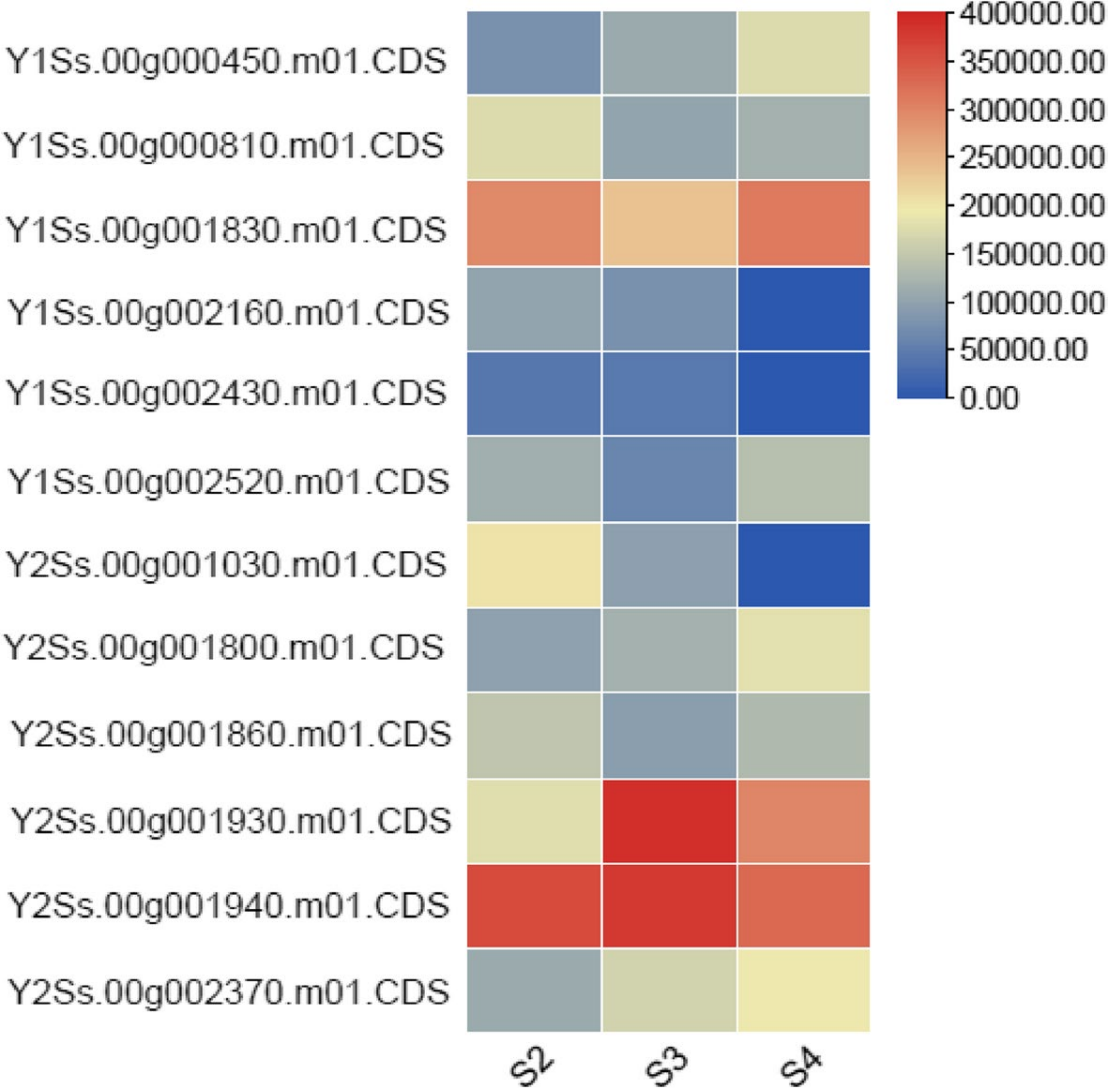
Heat map showing the expression level of 12 male-specific regions of male specific chromosome regions (Y1 and Y2) at different development stages (S) of the flowers (S2, S3, S4)

Out of 838 annotated genes found in the male specific regions of the Y chromosome, only 51 were unique to the male genome, of which 32 and 19 belonged to Y1 and Y2, respectively (Table 3). None of these genes matched in any form with either the female genome or the X chromosome, meaning that they are completely male-specific. All the 51 genes matched specifically to the Y1 and Y2 region of the Y chromosome (Fig. 2). The RNA-seq reads of the S2, S3 and S4 stages of male and female flowers were mapped to the CDS of the 51 male-specific genes and expression analysis was performed. The results showed that, in total, 32 genes were expressed in flowers, of which 19 and 13 belong to Y1 and Y2, respectively (Table 3; Table S5). Out of 32 genes, only 12 genes were highly expressed (> 80 reads) in all 3 flowering stages of the male and of these 6 and 6 belonged to Y1 and Y2 regions respectively (Fig. 3). The list of these 12 highly expressed male-flowering genes is given in (Table 4). The Paannzer2 (Protein ANNotation with Z-scoRE) [28] was used to understand the Gene Ontology (GO) annotations and predictions (Table S6). SANSparallel (SANS2) [29] analysis was performed as a high-performance homology search (Table S6). The results revealed only 3 genes with ontology description, Y1Ss.00g002520 encoding a CCHC-type domain-containing protein, Y2Ss.00g001860 a Adenylate isopentenyltransferase and Y2Ss.00g001930 a Zinc finger MYM-type protein 1 gene, while the others were novel and uncharacterized proteins. The gene (CDS) Y2Ss.00g001940, which was a novel uncharacterised protein, showed the highest expression level in male flowers, mostly affecting male flower development (Table 4; Table S5).

**Table 3:** Expression of male-unique genes from the Y1 and Y2 regions of chromosome 9. The jojoba male-specific genes do not exist in any form in the female genome

Chr Y (9) male-specific region	Total number of genes annotated	Number of genes (CDs) unique to the male genome	Number of genes (CDs) expressed in flowers	Number of genes not expressed in flowers (No reads mapped)	Number of lowly- expressed genes (< 20 reads)	Number of intermediate- expressed genes (20–80 reads)	Number of highly- expressed genes (> 80 reads)
Y1	344	32	19	13	9	4	6
Y2	494	19	13	6	4	3	6
Total	838	51	32	19	13	7	12

The expression level was calculated as the total number of reads mapped per gene (CDS) at three flowering stages (S2, S3, S4) for five biological replicates. The unit for expression profile is number of mapped reads-coverage (per CDS of the gene)

**Table 4:** Expression of unique male-specific genes from the Y1 and Y2 regions of chromosome 9 (mean value of S2, S3 and S4 stages (all stages)). The values were measured by StringTie using mapping files. All values for female genes were zero because no reads were mapped to these male-specific genes

Gene	Coverage	FPKM	TPM	Expression level in male	Expression level in female	Description by Paannzer2 and SANS2
Y1*Ss.00g000450	6.4	121,420	79,667	High	Not expressed	Uncharacterized protein
Y1Ss.00g000810	7.23	133,662	80,933	High	Not expressed	Uncharacterized protein
Y1Ss.00g001830	13.8	281,859	167,847	Very high	Not expressed	Uncharacterized protein
Y1Ss.00g002160	6.25	89,776	57,317	Medium	Not expressed	Uncharacterized protein
Y1Ss.00g002430	3.15	43,432	25,449	Medium	Not expressed	Uncharacterized protein
Y1Ss.00g002520	7.1	105,904	87,723	High	Not expressed	CCHC-type domain-containing protein; GO:0008270
Y2*Ss.00g001030	7.15	150,051	72,192	High	Not expressed	Uncharacterized protein
Y2Ss.00g001800	7.8	134,342	89,535	High	Not expressed	Uncharacterized protein
Y2Ss.00g001860	7.4	124,424	79,745	High	Not expressed	Adenylate isopentenyltransferase 3; GO:0009691
Y2Ss.00g001930	19.31	290,609	200,741	Very high	Not expressed	Zinc finger MYM-type protein 1
Y2Ss.00g001940	22.45	357,353	236,402	Very high	Not expressed	Uncharacterized protein
Y2Ss.00g002370	9.62	156,971	108,645	High	Not expressed	Uncharacterized protein

^*^The genes labelled as Y1 and Y2 were from the Y1 and Y2 regions of chromosome 9, respectively

### Differential express genes fold-change-specific enrichment analysis

Fold-Change-Specific Enrichment Analysis (FSEA) uses fold-change values from a transcriptome dataset to determine whether DEGs responding within specific fold-change ranges are enriched with specific GO classes. The FSEA was applied to the fold-change values from up and down regulated genes of male relative to female flowers in jojoba. FSEA detected 100 up-regulated (Table S7) and 42 down regulated (Table S8) GO terms respectively. The most significant FSEA of up-regulated and strongest quantiles based on a FSEA chart (Table 5, Figure S7) were GO:009733, GO:0006996, and GO:0009570 which represent the response to auxin, organelle organization and plastid stroma respectively. The strongest quantiles based on FSEA chart of down-regulated were GO:0006950, GO:0009628, and GO:0050896 which represent response to stress, response to abiotic stimulus, and response to stimulus (Table 6, Figure S7).

**Table 5:**
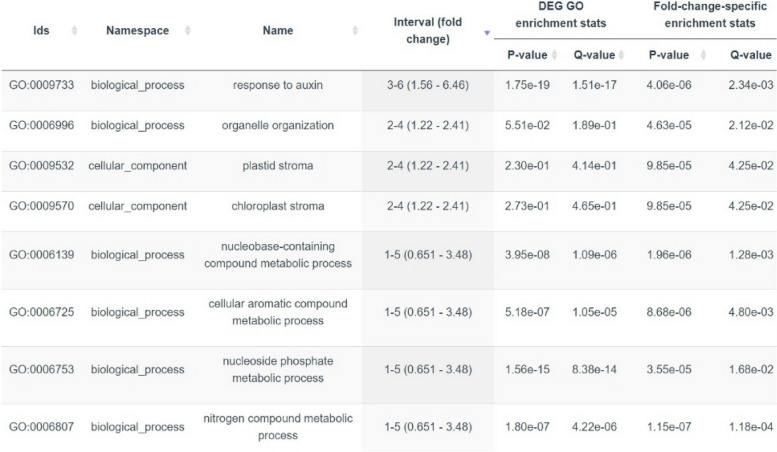
The most significant Fold-change-Specific Enrichment Analysis (FSEA) of up-regulated DEGs GO terms

**Table 6:**
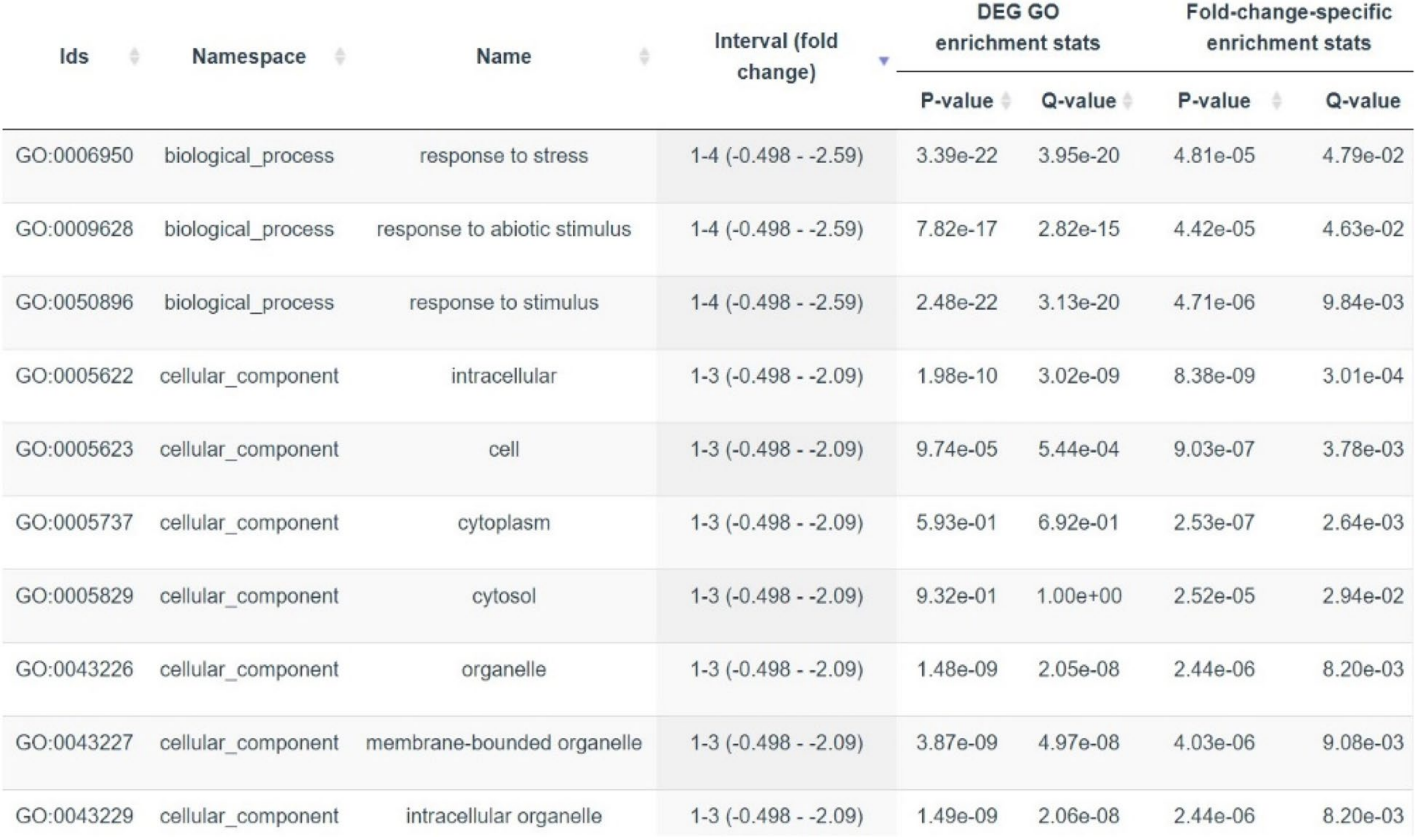
The most significant Fold-change-Specific Enrichment Analysis (FSEA) of down-regulated DEGs GO terms

## Discussion

The study of the differential expression of male and female jojoba flowers revealed an important finding for this desert plant. The most closely related sequences based on the functional annotation analysis for the coding sequences were from beetroot (*Beta vulgaris subsp. vulgaris*), quinoa (*Chenopodium quinoa*), and spinach (*Spinacia oleracea*). These plants share the same order, Caryophyllales, with Jojoba (*Simmondsia chinensis*). The DEG analysis shed more light on the genes that are associated with the flowers from the comparison between the male and female groups. A total of 16,923 significantly DEGs showed that a large number of genes were associated with sex specific flower formation. Transcription factors played an important role in the regulation of the flowering in jojoba plants. A total of 19 differentially expressed TFs families were detected which were involved in flower and pollen development. Among the various families, the MYB, ERF, bHLH families were the most abundant. The MYB protein family has been identified as being involved in abiotic stress responses [30], plant growth [31], and hormone signal transduction [32]. The MYB genes have also been identified as regulators of flower development [33]. A total of 69 differentially expressed MYBs were identified (Table S3) of these, MYB97/101 was identified as a male factor that controls pollen tube-synergid in *Arabidopsis thaliana* plant [34], MYB35/80 was associated with floral development and sex determination in *Cannabis sativa,*and MYB25 was required for male gametophyte (pollen) development [35]. Ethylene-responsive factors (ERFs) are found widely in the plant kingdom and include APETALA2/ Ethylene-responsive factors (AP2/ERE) which are involved in various processes in plants such as flowering time control, plant morphogenesis, and stress response [36, 37]. A total of 50 DEGs were associated with ERR and AP2/ERF (Table S3). The ERF RAP2.3, and RAP2.4 genes were expressed in flowers, leaves, stems, and roots in *Arabidopsis thaliana*plant [38]. The TF bHLH family has been found to regulate different flower development processes [39]. A total of 47 DEGs were associated with the bHLH family (Table S3) and among these, bHLH89/91 was associated with anther and pollen development in *Cannabis sativa* [7]. The bHLH 89 gene was also found to be associated with anther development and bHLH66/75 was reported to participate in flowering time and hormonal regulation in *Asparagus officinalis* [8].

Plant hormones (phytohormones) have an important role in regulating various aspects of plant growth and development including in the reproductive parts of plants [40]. Many studies have found that floral development and sex expression can be affected by various hormones including auxin, cytokinin, gibberellin, abscisic acid, and jasmonic acid [41–43]. Several plant hormone families were associated with DEGs in jojoba flowers analysis. Among the various hormone families, auxin, gibberellin, and abscisic acid families were the most abundant in the jojoba plant. Auxin is a fundamental molecule that controls many aspects of plant development and the response genes for auxin are classified into three families: small auxin up RNA (SAUR), auxin/indole-3-acetic acid (AUX/IAA) and gretchenhagen3 (GH3) [44]. Auxin was the hormone with the highest number of associated DEGs among the families with 73 DEGs among which 26 genes were linked with *SAUR* and 7 genes with AUX/IAA (Table S4). Gibberellin had the second highest number associated with 19 DEGs. The gibberellic acid-stimulated Arabidopsis (GASA) are a gene family found in different plants such as arabidopsis, rice*, *tomato, and petunia. A total of 7 genes of GASA including gibberellin-regulated protein 1 (GASA1), gibberellin-regulated protein 2-like (GASA2), gibberellin-regulated protein 4 (GASA4), gibberellin-regulated protein 6-like (GASA6), gibberellin-regulated protein 8 (GASA8), gibberellin-regulated protein 10 (GASA10), and gibberellin-regulated protein 14 (GASA 14) were DEGs in jojoba. All these genes found to be active in root, meristem, flower and seed tissues in Arabidopsis*. *Gibberellin-regulated protein 4 (GASA4) has a demonstrated role in floral meristem regulation and floral organ identity, prompting the size of the seeds and weight in mutant transgenic plants [45]. Gibberellin 20 oxidase 2 was found to be a DEG between male and female jojoba flowers and a key oxidase enzyme in the biosynthesis of gibberellin which is involved in the promotion of the floral transition, fertility and silique elongation in *Arabidopsis* [46]. Abscisic acid had the third highest number of associated genes, with 17 DEGs in jojoba. The abscisic acid 8'-hydroxylase 4 (CYP707A4) gene was reported to be mainly expressed in flowers with low expression in other tissues including leave, stem, and root [47]. The abscisic acid 8'-hydroxylase 1-like (CYP707A1) gene was also mainly expressed in flower, root and stem in Arabidopsis [48]. Both genes were DEGs in jojoba plant (Table S4).

The result of expression of male-specific genes (Y1 and Y2) analysis revealed 12 genes were highly expressed in all 3 flowering stages of the male. The transcriptome factors and plant hormones have critical roles in floral developments [49, 50] and the three known highly expressed genes found in male-specific parts were associated with transcriptome factor and plant hormone genes. Both CCHC type and MYM type protein 1 belong to the zinc finger transcriptome factor family. Zinc finger, CCHC-type has an important role in floral morphology and flowering initiation in *Monotropa hypopitys* and *Arabidopsis thaliana* and was also found to be linked with flower sex in the dioecious plant Yam (*Dioscorea spp.)* [51]. The third known gene found to be highly expressed in male-specific was Adenylate isopentenyltransferase which is involved in cytokinin biosynthesis. The analysis of functional enrichment was applied to the up and down regulated DEGs of male and female flowers samples. The highest enrichment of up regulated genes was response to auxin (GO:0009733). Auxin plays important role in flower development from initial growth to final stages of reproduction. Based on our analysis many DEGs were associated with plant hormones and the highest number of DEGs hormone genes was for auxin (Table S4). In addition, the highest number of upregulated DEGs in KEGG pathways were associated with plant hormone signal transduction (Table S2). The GO:0009733 corresponds to response to auxin which refers to the set of molecular events that occur in plant cells upon exposure to auxin hormone. The highest number of genes were found to be enriched in response to auxin from flowering and hormone related GO in cymbidium orchids [52]. The response to stress (GO:0006950) was the most significant FSEA of down-regulated DEG in jojoba. The response to stress (GO:0006950) also has down-regulated DEGs enriched of both rice [53] and *Arabidopsis thaliana* [54] plants. Jojoba is a desert plant and to survive the harsh condition many mechanisms of drought stress require downregulation of functions such as stomatal closure and decreased Rubisco activity [55]. The three highest down-regulated DEGs enriched were abiotic responses including response to stress (GO:0006950), response to abiotic stimulus (GO:0009628), and response to stimulus (GO:0050896) (Table 6, Figure S7).

## Conclusions

In this study, RNA-Seq and differential expression analysis in jojoba male and female flowers was conducted for the first time. The genes in the sex specific parts of the genome may be expressed differentially in other tissues but the flowers of the male and female are the most distinctly different features of the plants and the results confirm that the sex specific genes are very differently expressed in the flowers. The result showed a larger number of up-regulated than down-regulated genes between male and female flower groups indicating that male flower development requires more gene activation than suppression in the jojoba plant. These genes include different families of transcription factors that are associated with reproductive organs in the plant along with many plant hormone families that play an important role in flowering and pollen development. The 12 most highly expressed genes found in male specific parts included novel genes and known genes that are associated with flower development. These genes will be a valuable resource for breeders to use in efforts to manage the male biased ratio in jojoba plantings through plant biotechnology. Performing co-expression network analysis between the flower buds of continuous stages in both jojoba sexes may be valuable in future work. These results also contribute to our understanding of the control of flower development in jojoba and other dioecious plants.

## Materials and methods

### Plant materials

The flowers of mature male and female Jojoba plants were collected from “Chris-Egan” farm at Inglewood (151°4'0.20"E, 28°25′13"S) Queensland, Australia in September 2020.

### Sample preparation and RNA extraction

**Fig. 4:**
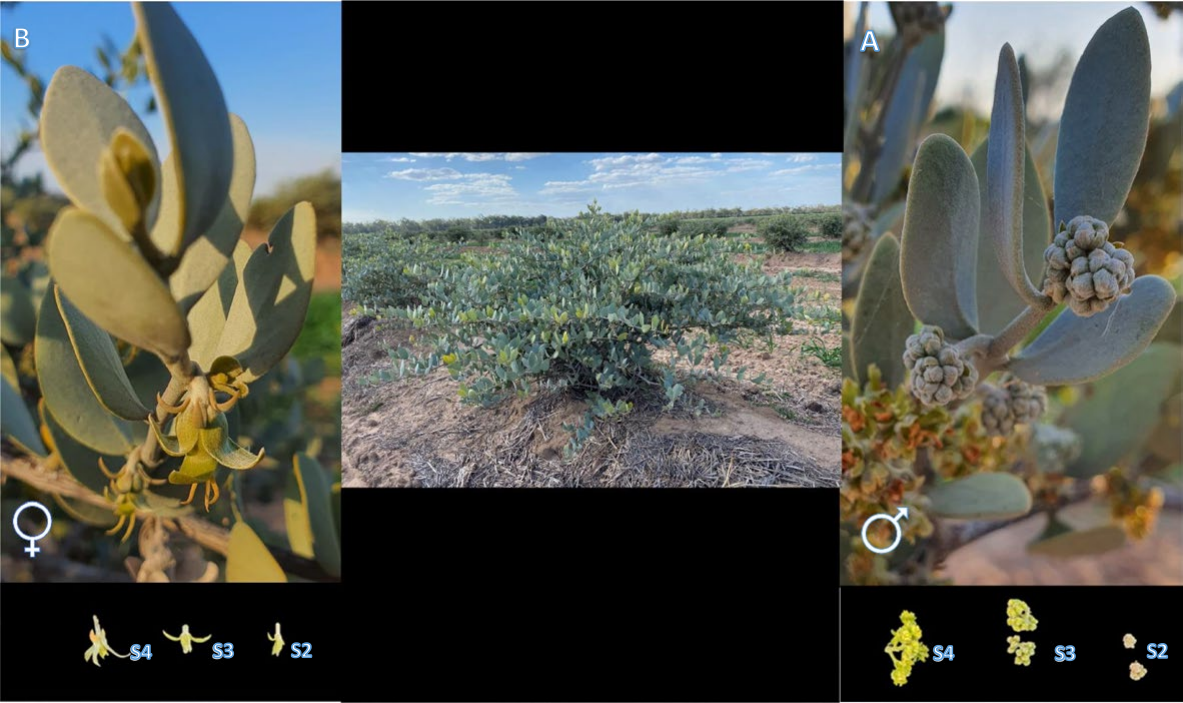
Illustration of the jojoba shrub and flowers of mature male and female Jojoba plants at Chris-Egan farm, Inglewood, Australia. **A**. Three different stages (S) of male (Dadi Dadi) jojoba plant. **B**. Three different stages of female (Wadi Wadi) jojoba plant

The developing flowers of five different plants from Dadi Dadi (male) and Wadi Wadi (female) were harvested in the field (Fig. 4). Three developmental stages (S) of flowers S2, S3, S4 were collected based on the different floral stages chart [10] from each individual plants resulting in 30 samples (15 male and 15 female). These samples were snap frozen in liquid nitrogen and placed in dry ice followed by preservation at − 80 ◦C in the laboratory prior to RNA extraction. The flower tissues were ground to a fine powder using a Tissue Lyser-II (Qiagen, US) at a frequency of 30/S for 30 s prior to RNA extraction. The RNA of the reproductive organs of male and female Jojoba plants was isolated using a two-step protocol including a cetyl trimethylammonium bromide (CTAB) method followed by a Qiagen RNeasy Plant mini kit (#74,134, Qiagen, Valencia, CA, United States) to assure the complete removal of the contaminating genomic DNA [56]. The qualitative and quantitative evaluation of the total RNA extracts were accomplished using a NanoDrop8000 spectrophotometer (TermoFisher Scientific, Wilmington, DE, USA) and 2100 Agilent Bioanalyzer (Agilent Technologies, Santa Clara, CA, USA), respectively. The RNA Integrity Number (RIN) is an algorithm for assigning integrity values to RNA measurements and was applied to analysis of the 30 samples of male and female RNA. The RIN number for the 15 male samples ranged from 4.4 to 7.5 and 6.30 to 7.70 for the 15 female samples.

### RNA-Seq and differential expression analysis

Equal amounts of RNA (35 µL) from 30 male and female flower samples were precipitated using sodium acetate and absolute ethanol before shipping to Macrogen Oceania (Bella Vista, NSW 2153, Australia) for sequencing. The libraries of all RNA samples were prepared using the TruSeq Stranded Total RNA Library Preparation Kit with Ribo-Zero Plant (Illumina Inc., San Diego, CA, USA) and then sequenced with an Illumina NovaSeq 6000 sequencing system. RNA-Seq analysis was performed using CLC Genomic Workbench software (CLC-GWS) (CLC Genomics Workbench. 11.0, http:// www. clcbio. com). Raw sequence data from the 30 samples of male and female jojoba flowers were quality trimmed (0.01). The trimmed reads were mapped to the reference of the CDS of the annotated Jojoba male genome from improved phase assembly (IPA) (13). The RNA-Seq reference was functionally annotated using OmicsBox (BioBam, Spain). These RNA-Seq data sets were used to identify differentially expressed (DE) transcripts associated with flower genes. The RNA from three different flower stages (S) (S2, S3, and S4) from each of Dadi Dadi (male) and Wadi Wadi (female) were collected for RNA-Seq analysis. Each three different stage of floral development were sampled in five replications. The data from RNA samples of three different stages of each replicate (R) were combined for RNA-Seq analysis for example for male (M) MR1S2, S3, S4 to MR5, S2, S3, S4 and for female (F) FR1S2, S3, S4 to FR5, S2, S3, S4 (5MR) and (5FR). The RNA-Seq analysis settings were; one reference sequence per transcript, mapping settings as; mismatch cost (2), insertion/deletion cost (3 each), length and deletion fraction (0.8 each), global alignment (no), auto-detect paired distances (yes), strand specific (both). The differential expression (DE) analysis for RNA-Seq male and female samples was also conducted using CLC-GWB. The library sizes were normalized using (TMM) trimmed mean of M-values method based on RNA-Seq normalization from the CLC-GWB manual. A metadata table was generated to specify the differently expressed transcripts between male and female groups. The metadata table was employed to create principal component plots to observe clustering of samples based on the average log2 (fold change) (log2FC) for the divergent transcripts between each group. The five male combined samples (MR1S2, S3, S4 to MR5, S2, S3, S4) were analysed as one male group. Similarly, the five female samples (FR1S2, S3, S4 to FR5, S2, S3, S4) were analysed as one female group to capture all the genes that were differentially expressed between the male and female throughout flowering. The differential expression involved the comparison of the female group against the male group where the female group was the control group to test differential expression due to sex. Significant differentially expressed (DE) transcripts for male and female groups were identified with an adjusted false discovery rate (FDR) *p*-value of ≤ 0.01. DEG analysis was performed using CLC-GWS were subjected to functional annotation using OmicsBox.

### Identification of male-specific genes in Y1 and Y2 and DEG analysis

**Fig. 5:**
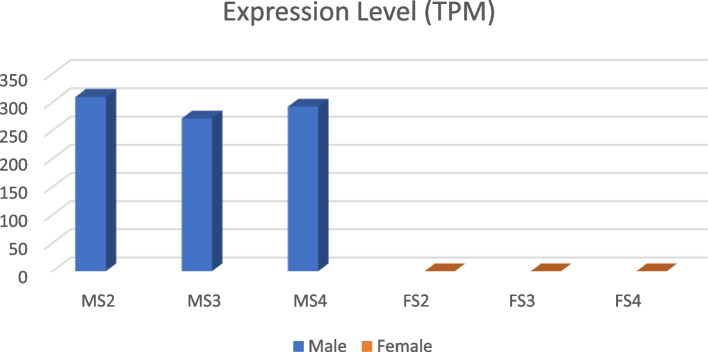
The average expression level of transcripts per million (TPM) of 32 expressed male-specific genes in Male (M) vs Female (F) flowers at S2, S3 and S4 stages of flowering. The expression level is zero in all flowering developmental stages of female due to the lack male-specific Y1 and Y2 regions/genes in the female genome

To be able to find unique male-specific genes, the DNA sequence of Y1 and Y2 regions were each annotated separately using both Augustus V3.4.0 and GeneMarkES V4.48 in the Genome Sequence Annotation Server (GeneSAS) annotation pipeline [18]. The CDSs of annotated Y1 and Y2 genes were mapped against the female genome (IPA assembly) using minimap2 and then the unmapped CDS were selected. These unmapped CDSs represented the genes that were present only in the male (male-specific). To confirm the high accuracy of the mapping process, the CDS of these male-specific genes were mapped against the female and male assembles (genomes). The CDS showed no match to female, while all the CDSs mapped to chromosome 9 (Y chromosome) of the male genome (Fig. 5) validating the accuracy of mapping and identity of male-specific genes. A standard differential expression analysis was conducted using the male-specific CDSs as a reference and RNA-seq data of male and female flowers at S2, S3, and S4 stages. For this reason, the RNA-seq data of male and female flowers (S2, S3, and S4 stages) were mapped against the reference (male-specific CDSs) using HISAT2 V2.2.1. The aligned read statistics were examined and then visualised in JBrowse to assess expression differences between male and female samples. The results of bam files from HISAT2 were then fed into StringTie [57] to counts the number of the reads and calculation of transcripts per million (TPM) and fragments per kilobase of transcript per million fragments mapped (FKPM) for transcripts to the male-specific CDSs. Subsequently, StringTie-merge was used to assembles the transcripts of corresponding replications into a non-redundant set of transcripts and to generate a combined count matrix. Finally, DESeq2 [58] was used to generate a list of differentially expressed genes. However, because the male-specific genes do not exist in female, any significantly expressed gene recognised be DESeq and StringTie could be considered as differentially expressed (Fig. 5). We used a combination of TPM, FPKM (Table S5) and median count read to divide the expression level of genes into three categories: low, intermediate and high with median count read of < 20, 20–80 and > 80, respectively (Table 7). Fold change specific enrichment analysis was performed using the tool at.https://webfsgor.sysbio.cytogen.ru/ [59].

**Table 7:** Expression of unique male-specific genes from the Y1 and Y2 regions of chromosome 9 (mean value of S2, S3 and S4 stages (all stages)). The values were measured by StringTie using mapping files. All values for female genes were zero because no reads were mapped to these male-specific genes

Gene	Coverage	FPKM	TPM	Expression level in male	Expression level in female	Description by Paannzer2 and SANS2
Y1*Ss.00g000450	6.4	121,420	79,667	High	Not expressed	Uncharacterized protein
Y1Ss.00g000810	7.23	133,662	80,933	High	Not expressed	Uncharacterized protein
Y1Ss.00g001830	13.8	281,859	167,847	Very high	Not expressed	Uncharacterized protein
Y1Ss.00g002160	6.25	89,776	57,317	Medium	Not expressed	Uncharacterized protein
Y1Ss.00g002430	3.15	43,432	25,449	Medium	Not expressed	Uncharacterized protein
Y1Ss.00g002520	7.1	105,904	87,723	High	Not expressed	CCHC-type domain-containing protein; GO:0008270
Y2*Ss.00g001030	7.15	150,051	72,192	High	Not expressed	Uncharacterized protein
Y2Ss.00g001800	7.8	134,342	89,535	High	Not expressed	Uncharacterized protein
Y2Ss.00g001860	7.4	124,424	79,745	High	Not expressed	Adenylate isopentenyltransferase 3; GO:0009691
Y2Ss.00g001930	19.31	290,609	200,741	Very high	Not expressed	Zinc finger MYM-type protein 1
Y2Ss.00g001940	22.45	357,353	236,402	Very high	Not expressed	Uncharacterized protein
Y2Ss.00g002370	9.62	156,971	108,645	High	Not expressed	Uncharacterized protein

^*^The genes labelled as Y1 and Y2 were from the Y1 and Y2 regions of chromosome 9, respectively

### Annotation of RNA-Seq reference

The coding sequence (CDS) from the annotation of male genome [13] was used as a reference for RNA-Seq analysis. To obtain a complementary CDS reference, RNA samples from jojoba leaves [13] and one replication of each RNA samples from different flower stages (S2, S3 and S4) from male and female plants were aligned with the reference male genome using HISAT2 V2.1.0. BRAKER2 V2.1.1 was chosen to predict protein-coding genes using the Genome Sequence Annotation Server (GeneSAS) annotation pipeline [18]. The Benchmarking Universal Single-Copy Orthologs BUSCO V5.3.2 was used for annotation completeness. The CDS were functionally annotated using a Basic Local Alignment Search Tool (BLAST) analysis with an e-value threshold of 1e-10 in OmicsBox V2.0. Gene Ontology (GO) terms that associated with the BLAST results were assigned to the transcripts to identify gene functions. The Kyoto Encyclopedia of Genes and Genomes (KEGG) pathway analysis was used to identify the significantly differentially expressed gene pathways. InterProScan also used with different families, domains, sites and repeats. The functional annotation of the CDS was used to identify all gene names of the significantly DE transcripts in OmicsBox.

### Supplementary Information

**Additional file 1:** **Figure S1.** Functional Annotation of the coding sequencing of jojoba male Jojoba genome (reference for RNA-Seq). (A) Benchmarking Universal Single-Copy Orthologs (BUSCO) for RNA-Seq reference. (B) Gene Ontology (GO) for the RNA-Seq’s reference. Illustration of the three GO categories Biological Process (BP), Molecular Function (MF) and Cellular Component (CC). The highest sequencing number linked with GO term of BP, MF, and CC were cellular process, binding, and cellular anatomical entity respectively.(C) Enzyme Code (EC) Distribution for the RNA-Seq’s reference. The highest three sequencings associated with EC classes were transferases, hydrolases and oxidoreductases. (D) Top-Hit Species Distribution based on blast analysis. The three highest species were *Beta vulgaris* followed by *Chenopodium quinoa* and *Spinacia oleracea.* **Figure S2.** Illustration of InterProScan Domain Distribution for transcriptome analysis reference. The InterProScan (IPS) Domain of the coding sequences (CDS) of male jojoba plant, which used as reference for RNA-Seq analysis. The three highest sequences associated with IPS domain were Protein kinase domain followed by Zinc finger Ring-type and RNA recognition motif domain. **Figure S3.** Illustration of both mapped and un-mapped percentage of male and female samples against the reference of coding sequences of male jojoba plant. The mapped percentage of male samples ranged between 52 to 55 % whereas the un-mapped percentage ranged between 45 to 49 %. The mapping percentage of female samples ranged between 51 to 55% and 44 to 48% for un-mapping reads. The pie charts are an example of mapping one male sample (R1MS2, S3, S4) and one female sample (R1FS2, S3, S4) against the reference. Male=M, Female=F, Replicate=R, Stage=S.** Figure S4.** Principal Component Analysis of the five male and female groups. The principal component 1 explained 51.9 % whereas principal component explained 12.8 %. The five female groups showed a tight cluster with each other whereas the five male groups showed a cluster in R1M S2, S3, S4, R2 MS2, S3, S4, and R3 M S2, S3, S4 and with a small distance with R4 M S2, S3, S4 and one outlier group R5 M S2, S3, S4. R=Replicate, M=Male, F=Female, S=Stage. **Figure S5.** Gene Ontology (GO) classification of up regulated significantly differentially expressed genes. Illustration of the three GO categories Biological Process (BP), Molecular Function (MF) and Cellular Component (CC). The highest sequencing number linked with GO term of BP, MF, and CC were cellular process, cellular anatomical entity, and binding respectively. **Figure S6.** Gene Ontology (GO) classification of down regulated significantly differentially expressed genes. Illustration of the three GO categories Biological Process (BP), Molecular Function (MF) and Cellular Component (CC). The highest sequencing number linked with GO term of BP, MF, and CC were cellular process, cellular anatomical entity, and binding respectively. **Figure S7.** The FoldGO output data for comparison of gene expression of male flower relative to female. The chart illustrates the fold-change intervals detected by Fold-change-Specific Enrichment Analysis (FSEA) where selected Gene Ontology (GO) terms (the whole list is in Table S7 and S8) displayed the most significant enrichment compared to the whole Differentially Expressed Genes (DEGs). The blue and yellow color represents the up and down regulated process of fold-change GO terms respectively.

**Additional file 2:** **Table S1.** The quality check and trimming details for raw RNA reads from 30 flower samples. **Table S2.** KEGG pathways of down regulated DEGs male flowers relative to female jojoba plants. **Table S3.** The top six transcription factors families (MYB, ERF, Bhlh, NAC, WRKY, MADS-box).** Table S4.** Plant hormone associated with flowering in Jojoba plant. **Table S5.** Coverage, FPKM and TPM of 12 male-specific genes of Y1 and Y2 regions (chr9) at S2, S3 and S4 stages. **Table S6 .** Gene ontology details of known genes of 51 male specific recognised by Pannzer2. **Table S7.** Total of 100 up- regulated GO terms detected by Fold-Change-Specific Enrichment Analysis (FSEA).**Table S8.**Total of 42 down- regulated GO terms detected by Fold-Change-Specific Enrichment Analysis (FSEA).

## Data Availability

The datasets of the RNA-Seq of male and female from jojoba flowers tissues that generated and/or analysed during the current study are available at the NCBI website under Bioproject ID PRJNA837637. Both assemblies of Jojoba male genome using Improved Phase Assembly and chromosome level assembly are available at the NCBI website under Bioproject ID PRJNA694450. The data is also available the Genome Warehouse website under BioProject number PRJCA006974.
