# Supplementary material for: Highly sex specific gene expression in Jojoba
Source: BMC Plant Biol. 2023 Sep 19;23:440. doi: 10.1186/s12870-023-04444-z (PMC10507870; doi:10.1186/s12870-023-04444-z)
Supplement: Supplementary file 1 — Additional file 1: Figure S1. Functional Annotation of the coding sequencing of jojoba male Jojoba genome (reference for RNA-Seq). (A) Benchmarking Universal Single-Copy Orthologs (BUSCO) for RNA-Seq reference. (B) Gene Ontology (GO) for the RNA-Seq’s reference. Illustration of the three GO categories Biological Process (BP), Molecular Function (MF) and Cellular Component (CC). The highest sequencing number linked with GO term of BP, MF, and CC were cellular process, binding, and cellular anatomical entity respectively.(C) Enzyme Code (EC) Distribution for the RNA-Seq’s reference. The highest three sequencings associated with EC classes were transferases, hydrolases and oxidoreductases. (D) Top-Hit Species Distribution based on blast analysis. The three highest species were Beta vulgaris followed by Chenopodium quinoa and Spinacia oleracea. Figure S2. Illustration of InterProScan Domain Distribution for transcriptome analysis reference. The InterProScan (IPS) Domain of the coding sequences (CDS) of male jojoba plant, which used as reference for RNA-Seq analysis. The three highest sequences associated with IPS domain were Protein kinase domain followed by Zinc finger Ring-type and RNA recognition motif domain. Figure S3. Illustration of both mapped and un-mapped percentage of male and female samples against the reference of coding sequences of male jojoba plant. The mapped percentage of male samples ranged between 52 to 55 % whereas the un-mapped percentage ranged between 45 to 49 %. The mapping percentage of female samples ranged between 51 to 55% and 44 to 48% for un-mapping reads. The pie charts are an example of mapping one male sample (R1MS2, S3, S4) and one female sample (R1FS2, S3, S4) against the reference. Male=M, Female=F, Replicate=R, Stage=S. Figure S4. Principal Component Analysis of the five male and female groups. The principal component 1 explained 51.9 % whereas principal component explained 12.8 %. The five female groups showed a tight cluster with [file 12870_2023_4444_MOESM1_ESM.zip › Figure S4.docx]

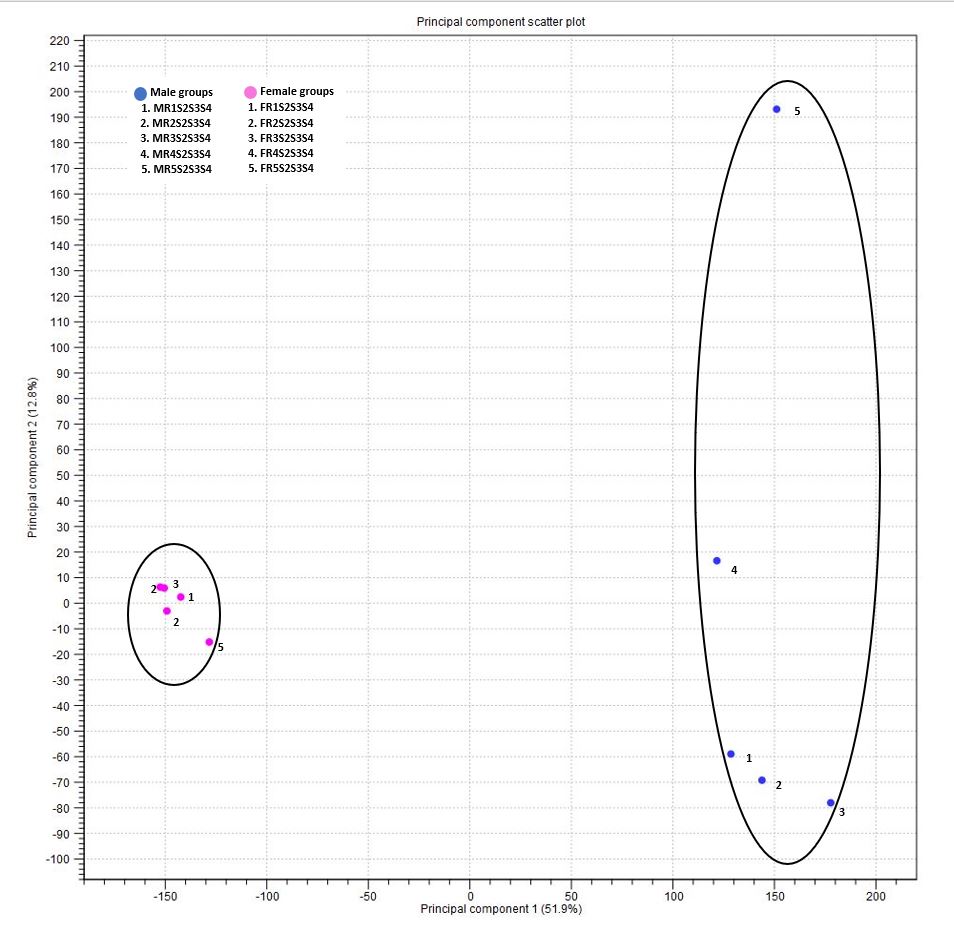


**Figure** S4. Principal Component Analysis of the five male and female groups. The principal component 1 explained 51.9 % whereas principal component explained 12.8 %. The five female groups showed a tight cluster with each other whereas the five male groups showed a cluster in R1M S2, S3, S4, R2 MS2, S3, S4, and R3 M S2, S3, S4 and with a small distance with R4 M S2, S3, S4 and one outlier group R5 M S2, S3, S4.

**R**=Replicate, **M**=Male, **F**=Female, **S**=Stage
