# Supplementary material for: Highly sex specific gene expression in Jojoba
Source: BMC Plant Biol. 2023 Sep 19;23:440. doi: 10.1186/s12870-023-04444-z (PMC10507870; doi:10.1186/s12870-023-04444-z)
Supplement: Supplementary file 2 — Additional file 2: Table S1. The quality check and trimming details for raw RNA reads from 30 flower samples. Table S2. KEGG pathways of down regulated DEGs male flowers relative to female jojoba plants. Table S3. The top six transcription factors families (MYB, ERF, Bhlh, NAC, WRKY, MADS-box). Table S4. Plant hormone associated with flowering in Jojoba plant. Table S5. Coverage, FPKM and TPM of 12 male-specific genes of Y1 and Y2 regions (chr9) at S2, S3 and S4 stages. Table S6 . Gene ontology details of known genes of 51 male specific recognised by Pannzer2. Table S7. Total of 100 up- regulated GO terms detected by Fold-Change-Specific Enrichment Analysis (FSEA).Table S8.Total of 42 down- regulated GO terms detected by Fold-Change-Specific Enrichment Analysis (FSEA). [file 12870_2023_4444_MOESM2_ESM.zip › Table S1.docx]

# Table S1. The quality check and trimming details for raw RNA reads from 30 flower samples

| **N** | **M & F Jojoba Seq ID** | **RAW # reads** | **RAW # bases** | **0.01 # reads** | **% Loss 0.01 reads** | **0.01 # bases** | **% Loss 0.01 bases** |
| --- | --- | --- | --- | --- | --- | --- | --- |
| **1** | **01 MS2R1** | **96,593,212** | **14,585,575,012** | **94,573,513** | **2.09** | **14,004,277,361** | **3.99** |
| **2** | **02MS2R2** | **88,297,684** | **13,332,950,284** | **86,411,348** | **2.14** | **12,807,299,708.00** | **3.94** |
| **3** | **03MS2R3** | **88,667,398** | **13,388,777,098** | **86,618,888** | **2.31** | **12,815,309,079.00** | **4.28** |
| **4** | **04MS2R4** | **105,082,766.00** | **15,867,497,666.00** | **102,965,358.00** | **2.01** | **15,258,679,549.00** | **3.84** |
| **5** | **05MS2R5** | **105,964,768.00** | **16,000,679,968.00** | **103,688,687.00** | **2.15** | **15,375,501,773.00** | **3.91** |
| **6** | **06FS2R1** | **98,527,046.00** | **14,877,583,946.00** | **96,341,364.00** | **2.22** | **14,271,170,324.00** | **4.08** |
| **7** | **07FS2R2** | **105,140,930.00** | **15,876,280,430.00** | **102,883,214.00** | **2.15** | **15,236,300,841.00** | **4.03** |
| **8** | **08FS2R3** | **94,449,688.00** | **14,261,902,888.00** | **92,247,659.00** | **2.33** | **13,665,248,105.00** | **4.18** |
| **9** | **09FS2R4** | **94,128,420.00** | **14,213,391,420.00** | **92,247,659.00** | **2.00** | **13,665,248,105.00** | **3.86** |
| **10** | **10FS2R5** | **96,841,354.00** | **14,623,044,454.00** | **94,796,120.00** | **2.11** | **14,051,678,373.00** | **3.91** |
| **11** | **11MS3R1** | **84,120,716.00** | **12,702,228,116.00** | **82,252,616.00** | **2.22** | **12,189,764,175.00** | **4.03** |
| **12** | **12MS3R2** | **92,243,734.00** | **13,928,803,834.00** | **90,330,595.00** | **2.07** | **13,387,519,697.00** | **3.89** |
| **13** | **13MS3R3** | **97,589,182.00** | **14,735,966,482.00** | **95,748,106.00** | **1.89** | **14,200,902,454.00** | **3.63** |
| **14** | **14MS3R4** | **99,781,962.00** | **15,067,076,262.00** | **97,653,884.00** | **2.13** | **14,469,739,169.00** | **3.96** |
| **15** | **15MS3R5** | **92,162,776.00** | **13,916,579,176.00** | **90,093,108.00** | **2.25** | **13,335,027,017.00** | **4.18** |
| **16** | **16FS3R1** | **105,125,594.00** | **15,873,964,694.00** | **102,867,785.00** | **2.15** | **15,240,086,721.00** | **3.99** |
| **17** | **17FS3R2** | **106,110,096.00** | **16,022,624,496.00** | **103,893,501.00** | **2.09** | **15,397,919,686.00** | **3.90** |
| **18** | **18FS3R3** | **100,471,120.00** | **15,171,139,120.00** | **98,127,426.00** | **2.33** | **14,509,489,332.00** | **4.36** |
| **19** | **19FS3R4** | **99,863,010.00** | **15,079,314,510.00** | **97,644,582.00** | **2.22** | **14,462,021,428.00** | **4.09** |
| **20** | **20FS3R5** | **100,512,320.00** | **15,177,360,320.00** | **98,209,307.00** | **2.29** | **14,553,980,617.00** | **4.11** |
| **21** | **21MS4R1** | **84,433,722.00** | **12,749,492,022.00** | **82,520,467.00** | **2.27** | **12,231,163,027.00** | **4.07** |
| **22** | **22MS4R2** | **93,365,362.00** | **14,098,169,662.00** | **91,189,260.00** | **2.33** | **13,495,434,277.00** | **4.28** |
| **23** | **23MS4R3** | **87,345,646.00** | **13,189,192,546.00** | **85,498,289.00** | **2.11** | **12,675,946,251.00** | **3.89** |
| **24** | **24MS4R4** | **105,174,906.00** | **15,881,410,806.00** | **102,995,554.00** | **2.07** | **15,273,572,604.00** | **3.83** |
| **25** | **25MS4R5** | **105,243,150.00** | **15,891,715,650.00** | **102,887,702.00** | **2.24** | **15,246,900,958.00** | **4.06** |
| **26** | **26FS4R1** | **89,397,072.00** | **13,498,957,872.00** | **87,349,840.00** | **2.29** | **12,939,540,016.00** | **4.14** |
| **27** | **27FS4R2** | **86,429,200.00** | **13,050,809,200.00** | **84,344,187.00** | **2.41** | **12,480,170,090.00** | **4.37** |
| **28** | **28FS4R3** | **105,676,768.00** | **15,957,191,968.00** | **103,262,826.00** | **2.28** | **15,293,405,527.00** | **4.16** |
| **29** | **29FS4R4** | **99,248,184.00** | **14,986,475,784.00** | **97,189,452.00** | **2.07** | **14,399,473,163.00** | **3.92** |
| **30** | **30FS4R5** | **105,152,564.00** | **15,878,037,164.00** | **102,917,048.00** | **2.13** | **15,246,542,929.00** | **3.98** |

**M**=male, **F**=female, **S**=stage, **R=**replication
